# Design and implementation of a high yield production system for recombinant expression of peptides

**DOI:** 10.1186/1475-2859-13-65

**Published:** 2014-05-07

**Authors:** Vida Rodríguez, Juan A Asenjo, Barbara A Andrews

**Affiliations:** 1 Centre for Biotechnology and Bioengineering (CeBiB), Department of Chemical Engineering and Biotechnology, University of Chile, Beauchef 851, Santiago, Chile

**Keywords:** *Escherichia coli*, Recombinant peptide production, Cell penetrating peptides, Peptide therapeutics, Thrombin cleavage

## Abstract

**Background:**

Making peptide pharmaceuticals involves challenging processes where many barriers, which include production and manufacture, need to be overcome. A non common but interesting research area is related to peptides with intracellular targets, which opens up new possibilities, allowing the modulation of processes occurring within the cell or interference with signaling pathways. However, if the bioactive sequence requires fusion to a carrier peptide to allow access into the cell, the resulting peptide could be such a length that traditional production could be difficult. The goal of the present study was the development of a flexible recombinant expression and purification system for peptides, as a contribution to the discovery and development of these potentially new drugs.

**Results:**

In this work, a high throughput recombinant expression and purification system for production of cell penetrating peptides in *Escherichia coli* has been designed and implemented. The system designed produces target peptides in an insoluble form by fusion to a hexahistidine tagged ketosteroid isomerase which is then separated by a highly efficient thrombin cleavage reaction procedure. The expression system was tested on the anticancer peptides p53pAnt and PNC27. These peptides comprise the C-terminal region and the N-terminal region of the protein p53, respectively, fused by its carboxyl terminal extreme to the cell penetrating peptide Penetratin. High yields of purified recombinant fused peptides were obtained in both cases; nevertheless, thrombin cleavage reaction was successful only for p53pAnt peptide release. The features of the system, together with the procedure developed, allow achievement of high production yields of over 30 mg of highly pure p53pAnt peptide per g of dry cell mass. It is proposed that the system could be used for production of other peptides at a similar yield.

**Conclusions:**

This study provides a system suitable for recombinant production of peptides for scientific research, including biological assays.

## Background

The study of peptides as therapeutic molecules has generated great interest in the pharmaceutical industry due to their low toxicity, high specificity and high biological activity [1]. However, there are still many challenges inherent to peptide production, particularly related to synthesis, scale-up and purification. At present the main method of peptide production is chemical synthesis. This involves sequential addition of each amino acid to complete the desired sequence, which leads to an accumulative reduction in performance [2,3]. Moreover, the extremely large consumption of solvents and production of waste, make it a non environmentally friendly method [3].

As an alternative synthesis method, recombinant production of peptides has become a good choice for production of long peptides (longer than 25 aa), mainly because it offers a good combination of cost-effectiveness, scalability and sustainability [4,5].

An interesting peptide research area is related to the study of therapeutic peptides with intracellular targets. Complex molecular mechanisms involved in the development of a disease are not limited to extracellular signaling. They also involve complex networks with intracellular regulation pathways which are worthy of exploration. However, this type of research has been limited, in part, by the low permeability of the cell membrane to most macromolecules [6]; less than 10% of peptide candidates entering clinical studies bind intracellular targets [7]. A common strategy is to fuse a bioactive molecule to a carrier peptide (Cell Penetrating Peptide, CPP). These are cationic peptides with the ability to get into the cells and it has been demonstrated that they can mediate intracellular delivery of covalently bonded molecules, amongst them bioactive peptide sequences [8-13]. In this case, the fusion usually results in a final peptide longer than 30 amino acids, making traditional production difficult. Despite this, currently, of the peptides in Phase II studies, there are three bioactive peptides that incorporate a CPP sequence to enter the cells [7]. Producing these peptides efficiently in order to minimize the production cost by means of a recombinant peptide production system, could make these peptides more attractive for the pharmaceutical industry.

Biosynthetic production of peptides in *Escherichia coli* by direct expression may lead to cytoplasmic degradation of the product, poor recovery yields or to generate host toxicity. To overcome these problems, expression systems often produce recombinant peptides as fused peptides, bound to a protein with specific properties [14,15] or as tandem repeats of the peptide sequence [16]. These strategies require a subsequent hydrolysis step to release the target peptide sequence. Usually, chemical hydrolysis on methionine residues, on Asp^Pro [15] or on Asn^Gly [16] with cyanogen bromide (CNBr), formic acid or hydroxilamine, respectively, is used. These reagents are highly toxic, so alternatively, enzymatic proteolysis can be used for separation by including specific recognition sequences of common proteases. However, the latter has the disadvantage of being more expensive than chemical proteolysis.

A noteworthy peptide production method is the pET-31b(+) vector system for *E. coli*, from Novagen. This allows cloning of the target peptide sequence downstream of a ketosteroid isomerase (KSI) coding sequence and upstream of a polyhistidine tag sequence. The KSI protein is highly insoluble, so directs the expression of the fusion protein to produce inclusion bodies, preventing polypeptide degradation during bacterial expression and the polyhistidine tag allows purification of the fused peptide by nickel affinity chromatography. The peptide sequence must be flanked with methionine residues and could be cloned as tandem repeated sequence, then the target peptide could be separated from the partner protein by chemical hydrolysis with CNBr. Finally, target peptides are recovered from the cleavage product by chromatographic separation. High peptide productivity of more than 50 mg per liter of a culture at a density of 2.5 g of cells per liter has been achieved using this method [17]. Nevertheless, the target peptide sequences are limited to those not containing methionine residues and the process involves working with a hazardous reagent. Moreover, chemical hydrolysis with CNBr leads to peptide monomers with a C-terminal homoserine lactone and could lead to chemical modifications on amino acid side chains on the target peptide [18]. Some attempts have been made to modify this system to, alternatively, liberate the target peptide through enzymatic reactions. However, these methods have required the use of high amounts of enzyme to achieve an efficient proteolysis process, increasing the production cost and thus, limiting their use [19,20].

The aim of this work is the design and implementation of a flexible method for an efficient recombinant bacterial expression and purification system for production of potentially therapeutic peptides with intracellular targets. The expression system developed in *Escherichia coli* has been designed to be flexible in expressing and purifying potentially therapeutic peptides fused to the cell penetrating peptide Penetratin (KKWKMRRNQFWVKVQRG) [21,22]. The system includes a fusion partner which comprises a hexahistidine-tagged ketosteroid isomerase domain and a thrombin cleavage site, in order to allow a high expression yield and facilitate purification, and to liberate the peptide from the KSI protein, respectively. Unlike the system described above, the procedure designed allows a high yield production process at a relatively low cost, avoiding the use of hazardous reagents.

The expression system was tested on the anticancer peptides p53pAnt and PNC27. The p53pAnt peptide comprises the C-terminal region of the protein p53, as bioactive sequence, fused by its carboxyl terminal extreme to the cell penetrating peptide Penetratin, as transporter sequence, (GSRAHSSHLKSKKGQSTSRH*KKWKMRRNQFWVKVQRG*). It has been shown that this peptide selectively induces apoptosis in cancer cells with mutated or overexpressed p53 through p53-dependent processes [23,24]. The anticancer peptide has been tested *in vitro* in different cell types, such as colon cancer cells [23,25], breast cancer [24,25], lung [25] and lymphoma [23]. Furthermore, it has been reported to selectively produce necrosis in prostate cancer cells [26] and that it is able to selectively induce cell death by apoptosis *in vivo*, in a model of brain tumor (glioma) in rats [27]. The PNC27 peptide comprises the mdm-2 binding domain of the p53 protein (residues 12–26) containing a C-terminal Penetratin sequence as carrier (PPLSQETFSDLWKLL*KKWKMRRNQFWVKVQRG*) [28]. Unexpectedly, this peptide was found to induce selective necrosis in various carcinogenic cell types [29-31], as it has an amphipatic structure [32,33] which allows it to integrate into the cell membrane forming pores, producing membrane disruption and cell death by a p53-independent mechanism [29,32,34]. The selectivity on tumor cells would be given by the presence of mdm-2 on the plasma membrane of these cells [35,36], which is related to the role of mdm-2 in E-cadherin ubiquitination and degradation [36]. Thus, in non-tumor cells, PNC27 enters the cell, but in cancer cells it is retained in the membrane due to its affinity for mdm-2, and forms pores [35].

This paper shows the design of a system for producing high yield fused recombinant peptides and a procedure for efficient enzymatic cleavage to liberate the target peptides, which allowed production of highly pure p53pAnt peptide. Although the system failed at the proteolysis step in PNC27 peptide production, it is proposed that it could be potentially used for the production of other peptides.

## Results

### Design and construction of a pET31HT expression vector

**Figure 1:**
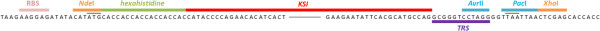
**Schematic diagram of the expression vector pET31HT.** Green: Polyhistidine tag coding sequence; red: ketosteroid isomerase gene; purple: thrombin recognition site coding sequence; light blue: cloning site restriction enzymes. Start and stop translation codons are crowned in brown.

Modifications made to pET-31b(+) allowed setting up the pET31HT vector. It comprises a KSI gene which contains an N-terminal hexahistidine tag-coding sequence to facilitate purification. A thrombin recognition site was positioned downstream of the KSI sequence, followed by *AvrII* and *PacI* restriction enzyme sites which compose the cloning site for the target peptide coding sequence. The *AvrII* site encodes the last two amino acids of the thrombin recognition site and the *PacI* site, which was designed to remain downstream of the cloned sequence, includes the stop codon for translation. Thus, the design allows cloning different potentially therapeutic cell penetrating peptides between these restriction enzyme sites and subsequently to separate them from the KSI fusion partner leaving no additional residues from the original peptide sequence. A schematic representation of the construct is shown in Figure 1.

### Expression and purification of recombinant fused peptides

**Figure 2:**
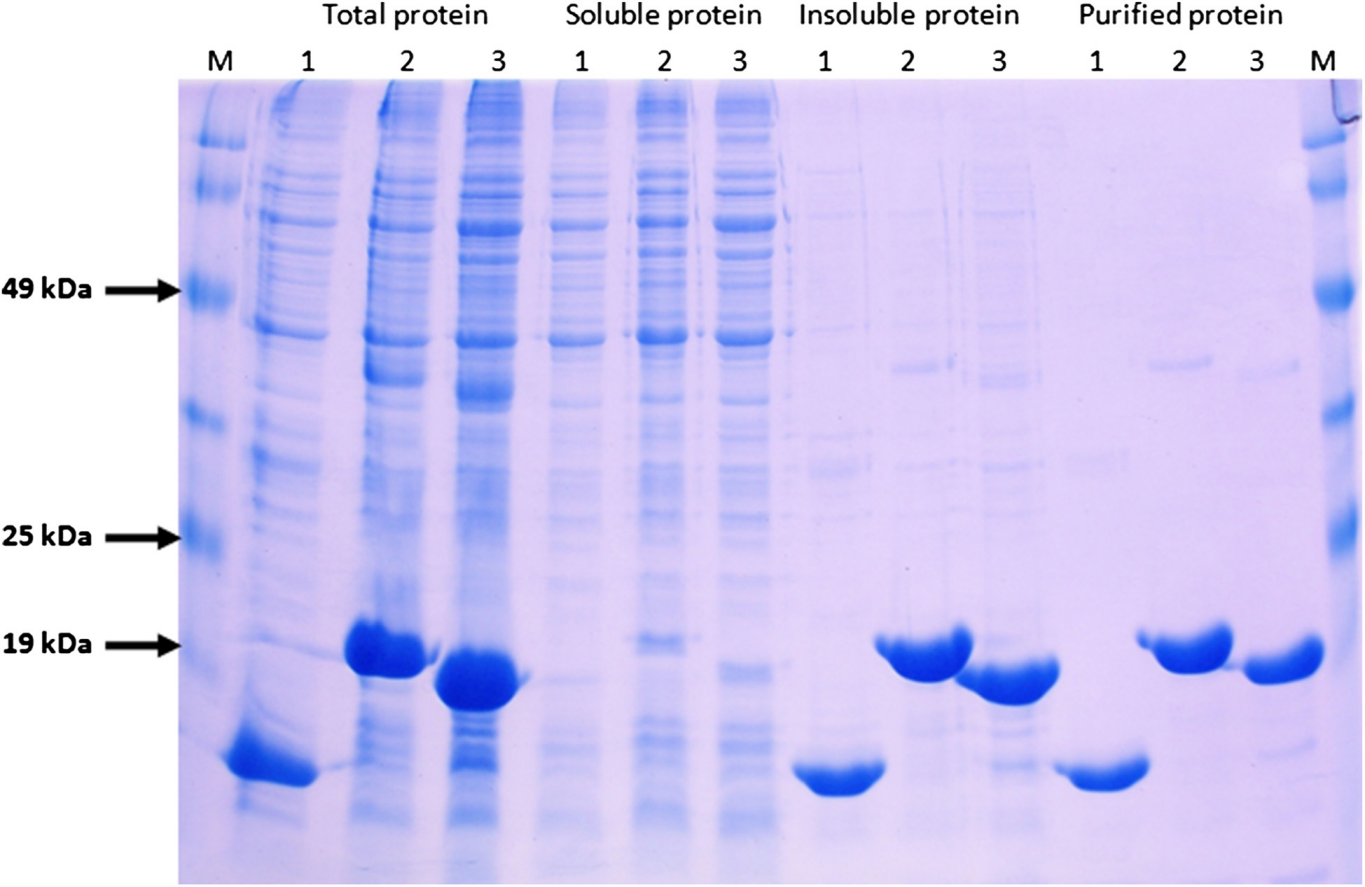
**SDS-PAGE analysis of recombinant protein expression in *E. coli *BL21(DE3).** SDS-PAGE 12.5% T, 3% C; Tris-Glycine buffer system. M: Molecular weight marker; 1: KSI (14.73 kDa); 2: KSI-p53pAnt (19.09 kDa); 3: KSI-PNC27 (18.69 kDa).

The peptide-encoding sequences were cloned into the expression vector pET31HT by the procedure described in *Methods*. The resulting vectors pET31HT-p53pAnt and pET31HT-PNC27, confirmed by DNA sequencing, were transformed into the expression host *Escherichia coli* BL21(DE3). A transformant colony of every construct was selected and grown at 37°C in LB medium containing 100 μg/mL ampicillin and induced by IPTG addition. A colony harboring the pET31HT vector was used as induction control. As shown in Figure 2 recombinant proteins KSI, KSI-p53pAnt and KSI-PNC27 were in an insoluble form and accounted for about 40% of the total protein. The productivity of the KSI control protein and the fused peptides was almost 20% of the dry cell mass. Recombinant proteins were purified from the insoluble fraction by Ni^+2^ affinity chromatography in denaturing conditions. About 95% of the recombinant protein was purified in a single elution fraction of equal volume to the loaded sample. It was observed that it is possible to recover all the recombinant protein bound to the resin by additional elutions of smaller volumes which then could be combined with the main elution. The purity of the eluted recombinant protein was estimated by SDS-PAGE analysis and it was determined to exceed 90% (Figure 2).

### Proteolytic cleavage and purification of recombinant peptides

**Figure 3:**
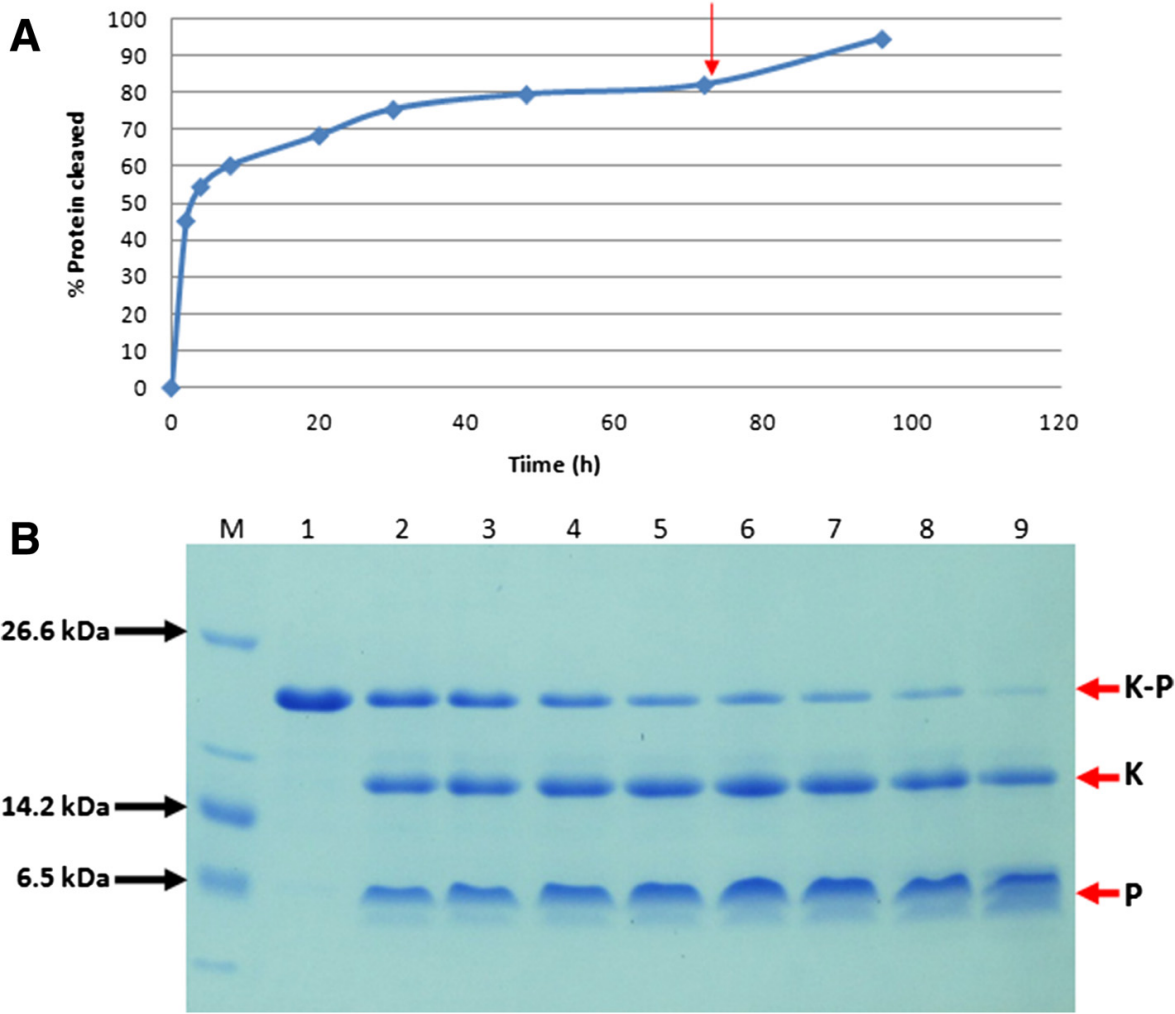
**Thrombin cleavage reaction progress at 23°C and 0.35 units of enzyme per mg of protein. A**: Reaction curve. The arrow indicates addition of an equal amount of thrombin. **B**: SDS-PAGE 10% T, 3% C; Tris-Tricine buffer system. Different species are indicated as K-P: KSI-peptide fused peptide; K: KSI partner protein; P: Peptide. M: Molecular weight marker; lanes 1–9: samples of the reaction at different times in hours. Lane 1: 0 h; lane 2: 2 h; lane 3: 4 h; lane 4: 8 h; lane 5: 20 h; lane 6: 30 h; lane 7: 48 h; lane 8: 72 h; lane 9: 96 h.

Precipitation of purified fused peptides through dilution allows recovery of practically all of the protein, with no loss detected in the supernatant by SDS-PAGE analysis. The precipitated protein was solubilized in cleavage buffer. Thrombin was added to the sample at a ratio of 0.35 unit of enzyme per mg of protein and incubated at 23°C with gentle shaking. Figure 3 shows the progress of the enzymatic cleavage reaction for p53pAnt fused peptide. Enzymatic cleavage of the fused peptide achieved about 60% digestion in 6 hours and about 80% digestion when the reaction was extended to 48 hours. When the reaction rate decreased and the curve became asymptotic, the addition of another equal amount of thrombin increased digestion to 95% of the fused peptide.

In the case of KSI-PNC27 recombinant protein, unexpectedly, incubation with thrombin protease did not cleave the fusion protein since no peptide hydrolysis product was detected on SDS-PAGE analysis.

The digestion product of KSI-p53pAnt was loaded onto a nickel-nitrilotriacetic acid (Ni-NTA) agarose column. The flow-through containing the p53pAnt peptide was collected. The column was washed with cleavage buffer to recover most of peptide in solution. A final wash with elution buffer was made in order to analyze the fraction retained in the column. As expected, the liberated KSI protein and the uncleaved KSI-p53pAnt fused peptide were retained by the column because both have a N-terminal polyhistidine tag. However, about 15% of the peptide was also bound to the column and was not eluted by the cleavage buffer wash. A summary of the purification procedure is shown in Table 1. Overall productivity obtained was more than 30 mg of peptide per g of dry cell mass.

**Table 1:** **Purification of KSI–p53pAnt fused peptide and p53pAnt peptide, overexpressed in *E. coli *** **BL21(DE3)**

**Purification step**	**Total fused peptide (mg)**	**Total p53pAnt (mg)**	**Purity (%)**	**Yield (%)**
Total protein extract	n.d	n.d	43*	100
Inclusion bodies	n.d	n.d	79*	-
First affinity chromatography	184**	43	90*	-
Precipitation recovery	184	43	-	100*
Thrombin cleavage	-	-	-	95*
Second affinity chromatography	-	34**	95	85*

Data per g of dry cell weight.

Experiments n.d., not determined.

*Determined by SDS-PAGE gels densitometry analysis.

**Determined by Bradford assay.

**Figure 4:**
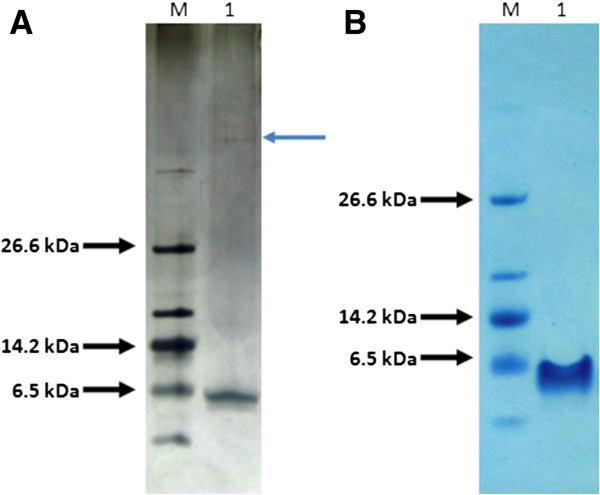
**Purified p53pAnt peptide.** SDS-PAGE 10% T, 3% C; Tris-Tricine buffer system. M: Molecular weight marker; 1: p53pAnt. **A**: 0.125 μg of peptide visualized with silver staining. Blue arrow indicates a contaminant protein band. **B**: 2 μg of peptide visualized with Coomasie staining. The contaminant protein band is not visualized.

The purity of the resulting peptide was determined on SDS-PAGE analysis visualized by silver staining and a high molecular weight contaminant protein was detected (Figure 4A). In order to estimate the ratio between the peptide and this protein by densitometry analysis, 2 mg of purified peptide were loaded onto a single lane on a second SDS-PAGE gel and was visualized by Coomasie staining, but the contaminant band was not detected (Figure 4B). Assuming that this band is right at the limit of detection of the Coomasie staining (0.1 μg for a single protein band [37]), the purity of the recombinant peptide was estimated as at least 95%.

**Figure 5:**
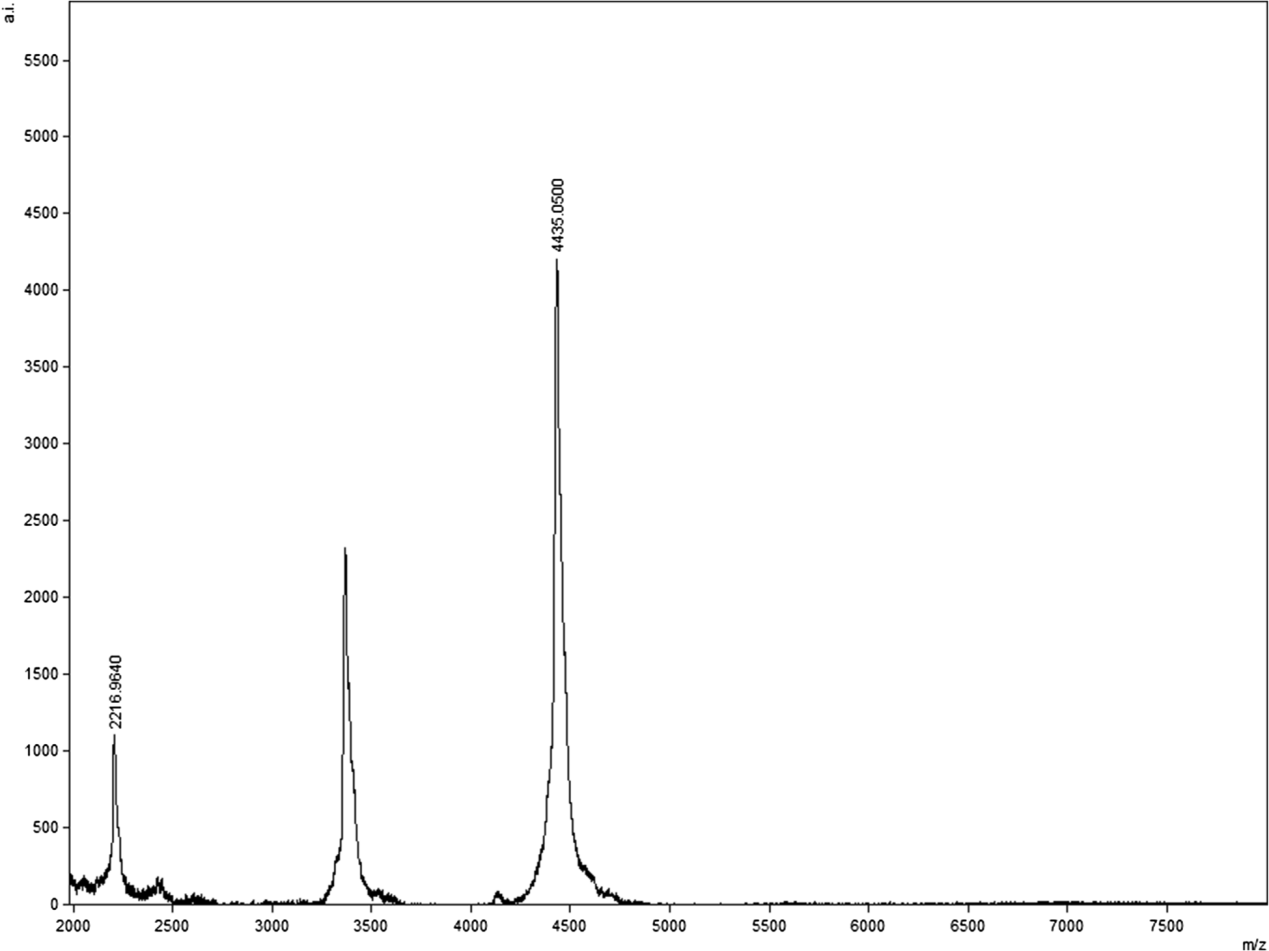
MALDI-TOF mass spectrum of the purified p53pAnt peptide.

A sample of the purified p53pAnt peptide was analyzed by Matrix Assisted Laser Desorption Ionization Time-of-Flight (MALDI-TOF) mass spectrometry. The theoretical m/z average ratio of the p53pAnt peptide is 4,435. The spectrum obtained contained the expected signal and the signal corresponding to the doubly protonated molecule (m/z = 2,218) (Figure 5), indicating that the resultant peptide actually corresponds to p53pAnt. No signal attributable to the detected contaminating protein shown in Figure 4A was detected.

## Discussion

The system presented in this paper was developed as an alternative tool to be used in research and development of new peptide drugs, especially for those with intracellular therapeutic targets. The procedure requires the attachment of the bioactive sequence to the cell penetrating peptide, Penetratin. This sequence allows entry into the cell and delivery to the intracellular target. The whole oligonucleotide sequence must be codon optimized and cloned in the designed vector.

In order to protect the peptides from enzymatic degradation during production and thus to achieve higher yields, the system directs the expression of the cloned peptide to produce inclusion bodies by its fusion to the insoluble protein ketosteroid isomerase. Inclusion bodies are stable against proteolysis and also have the advantage of being easily recoverable by centrifugation. Furthermore, they usually have high recombinant protein concentration and low relative amounts of contaminants. As a result, the procedure allows an initial recovery of the recombinant fused peptide with relatively high purity of more than 75% of the inclusion body protein content.

The system was designed to allow purification of the peptides attached to the KSI protein by immobilized metal affinity chromatography (IMAC) by including a polyhistidine tag. Then, the fused peptide construct is bound to the column and eluted, to later be subjected to enzymatic cleavage to release the target peptides. Finally, the digestion product is loaded onto a second IMAC chromatography, where the partner protein, the non-cleaved protein and any impurity present after the first chromatography will bind to the column, allowing obtention of a highly pure peptide in the flow-through.

Unlike other common systems that use KSI as a fusion protein partner for recombinant production of peptides, the designed system allowed production of peptide sequences without restriction on the presence of any amino acidic residue and without using acid conditions or toxic reagents such as hydroxylamine or cyanogen bromide. Instead, this system incorporates a thrombin recognition site strategically located so that the peptide is separated from the KSI protein without leaving any additional residues. Of common proteases, the lower cost of cleaving fusion proteins, per mg, is obtained using thrombin [15]. Moreover, the designed procedure for the hydrolysis reaction allowed recovery of the p53pAnt peptide from its fusion partner through a highly efficient enzymatic cleavage reaction. Sarkosyl was used to induce mild denaturing effects on protein structures, so its presence in the cleavage buffer allows maintenance of the fused peptide in solution in conditions compatible with thrombin activity, and inducing flexibility of protein structures enables exposure of recognition sites of the enzyme. These reaction conditions allow a 50% reduction of the amount of thrombin used to cleave one mg of fused peptide when compared to the recommendation of the supplier, and the use of at least 20 times less enzyme than the amount used in similar expression systems [19,20], since, as shown in Figure 3B, the procedure achieves cleavage of 2 mg of protein per unit of thrombin in 16 hours.

Although the enzymatic cleavage reaction failed for KSI-PNC27 protein, this result is not discouraging as it was probably due to the presence of proline in the thrombin cleavage site, which provides rigidity to the protein structure and does not allow proper coupling interaction of the protease in its recognition site, making the chosen peptide sequence not suitable for thrombin proteolysis.

It was observed that in the Ni-NTA chromatography step for after-cleavage peptide purification, the process requires a total protein concentration below 1 mg/mL before loading the sample onto the column. This step is conducted in mild denaturing conditions, therefore, not all the residues of the protein are exposed. This could lead to different concentration dependent conformations, in which at higher concentrations, the polyhistidine tag could not be exposed in all species of the dynamic equilibrium or either protein-protein/column-protein interactions could lead to steric hindrance, thus impeding the proteins to bind to the resin.

There was a loss of about 15% of the p53pAnt peptide that remains strongly attached to the resin during this last purification process, probably due to the three histidine residues of the bioactive peptide sequence as it was not eluted by salt or urea but only with high imidazole concentrations. It is postulated that this loss could vary depending on the histidine residue content of the target peptide.

Due to the use of thrombin, the system has some minor restrictions in the first and second amino acid of the bioactive sequence. As has been described in the literature [38], they cannot be acidic amino acids and, as discussed above, it was observed that Pro is also a restricted amino acid in these positions. With these exceptions, it is proposed that the system designed could provide a sustainable and efficient peptide manufacturing process, for almost any peptide sequence, since recombinant protein production and purification levels were very similar for KSI, KSI-p53pAnt and KSI-PNC27. Hence, the negative result in producing PNC27 peptide is considered inherent to its amino acid sequence rather than because of the designed procedure.

As recombinant protein is about 20% of dry cell mass in all cases, the potential productivity of p53pAnt and PNC27 was estimated from the relative ratio of the peptides size compared to the sequence of the recombinant KSI-peptide proteins. The p53pAnt peptide accounts for 23% of the fused peptide, while the PNC27 peptide comprises 21% of the recombinant KSI-peptide protein. Thereby, potential productivity of p53pAnt peptide is 46 mg per g dry cell weight and for PNC27 the calculated value is 42 mg per g dry cell weight.

From the values shown in Table 1, overall productivity of p53pAnt was estimated as 37 mg per g dry cell weight. Experimental results indicate that the overall productivity of p53pAnt is about 34 mg per g of dry cell mass, very close to the estimated value. In the case of PNC27, this estimation could not be done as the cleavage reaction was not successful. However, it is postulated that for the case of a generic peptide, which accounts for at least 20% of the recombinant KSI-peptide protein, 200 mg of total recombinant protein (KSI-peptide) per g cell dry weight could be produced. This would result in potential peptide productivity greater than 40 mg per g dry cell weight, and after the purification step a final productivity of over 30 mg per g dry cell weight of purified peptide. However, it should be noted that the final value will always depend on the particular properties of the sequence to be produced, especially, the amino terminal amino acids, which influence the efficiency of enzymatic proteolysis, and the number of histidine residues in the bioactive sequence, which could be important for peptide purification efficiency.

The peptide productivity obtained is high compared with that reported in other systems for recombinant peptide production [14-17,39-41]. The highest reported productivity was about 20 mg/g of cell [17], and in the present system, 30 mg of purified peptide per gram of cell could be obtained.

The cleavage procedure is the limiting step of the process. To make the process faster, a second dose of enzyme could be added at 16 hours of the reaction. On the other hand, if it is worth sacrificing overall productivity in exchange for a faster process, the reaction could be stopped at this time and the final productivity would not be less than 20 mg of peptide per gram of dry cell mass.

Finally, it was observed that, at different induction conditions, recombinant protein production remains at about 20% of the dry cell mass, hence, using fed-batch cultures of *Escherichia coli,* which enable obtention of 100 g/l of cells [42] could lead to a productivity of over 3 g/l of peptide.

## Conclusions

Recombinant fused peptides were produced as relatively pure protein in inclusion bodies and were purified by affinity chromatography under denaturing conditions. The urea-denatured protein was recovered by dilution precipitation and solubilized in a specific mild denaturing conditions buffer. The procedure designed allows separation of the peptide p53pAnt and the KSI partner protein by enzymatic proteolysis with thrombin, in a highly efficient manner; however, the proteolysis process failed to release PNC27. The recombinant p53pAnt peptide was purified from the cleavage product by Ni-NTA affinity chromatography, and recovered in the supernatant with a high purity, which was estimated to be at least 95%. The designed system has high productivity of more than 30 mg of purified peptide per gram of dry cell weight and is suitable for the production of peptides for scientific research, since it achieves the scale and purity required, even for biological assays used in therapeutic peptides research.

## Methods

*Escherichia coli* strains DH5α and BL21(DE3) were used as hosts for cloning and expression, respectively. The pGEM-T Easy vector (Promega) was used for cloning oligonucleotide sequences of the peptides. The pET-31b(+) vector (Novagen) was used as a template for construction of the modified expression vector pET31HT. Elongase enzyme (Invitrogen) was used for the PCR amplification of sequences. Restriction enzymes *NdeI, XhoI, PacI, AvrII* (New England Biolabs) and T4 DNA ligase (Invitrogen) were used according to the recommendation of the supplier. Thrombin, restriction grade, was purchased from Novagen. Oligonucleotides were synthesized by Integrated DNA Technologies. For protein purification, Ni-NTA agarose (QIAGEN) was used.

Proteins and peptides were loaded on SDS-PAGE gels for analysis, and concentration was determined by the Bradford assay, using bovine serum albumin as the standard. Densitometry analysis of SDS-PAGE gels was done using ImageJ software.

### pET31HT expression vector construction

To amplify the KSI sequence from the pET-31b(+) vector as a template, the following primers were designed and used: 5′-CATATGCACCACCACCACCACCACCATACCCCAGAACACA- 3′ and 5′-CTCGAGTTAATTAACCCCTAGGACCCGCCTGGCATGCGTGAAT- 3′. Thereby, the hexahistidine tag present in the template was moved from the C-terminal to the N-terminal of the KSI sequence and a thrombin cleavage sequence was introduced downstream of the KSI gene, followed by an *AvrII* site and a *PacI* site. These restriction enzyme sites allow cloning of DNA sequences coding potentially therapeutic cell penetrating peptides. The *NdeI* and *XhoI* cloning sites present in the original vector flanking the KSI gene, were kept at the N-terminal and C-terminal, respectively.

The PCR product was cloned into the pGEM-T Easy vector, and subcloned into pET-31b(+) expression vector between the *NdeI* and *XhoI* restriction enzymes sites.

### Cloning of the DNA sequences encoding the peptides

The DNA sequences encoding peptides were synthesized as two complementary oligonucleotides. The sequences include *AvrII* and *PacI* restriction enzyme sites to facilitate cloning into the pET31HT expression vector. The peptide-encoding DNA sequences were designed in order to avoid codons used with low frequency in *Escherichia coli*. To anneal the single strand oligonucleotides, they were heated at 95°C for 5 minutes, then left in a boiling water bath and allowed to cool slowly to room temperature. Dimers were adenylated and cloned into the pGEM-T Easy vector.

The final constructs were built from the sequences cloned into the pGEM-T Easy vector and the pET31HT expression vector, through the *AvrII* and *PacI* restriction enzyme sites.

The constructed plasmids pET31HT, pET31HT-p53pAnt and pET31HT-PNC27 were sequenced by Macrogen (Korea) and transformed into *Escherichia coli* BL21(DE3) electrocompetent cells.

### Production and purification of fused peptides

*Escherichia coli* BL21(DE3) harboring the expression vectors were cultivated in LB medium containing 100 μg/ml ampicillin, at 37°C with shaking. The overnight cultures were used to inoculate 100 ml LB media (with 100 μg/ml ampicillin) to an initial OD_600_ = 0.05, and grown at 37°C with shaking. When OD_600_ of the cultures reached 0.6, IPTG was added to a final concentration of 1 mM. After 3–6 h induction, cells were harvested by centrifugation, resuspended in binding buffer (40 mM Tris, 500 mM NaCl, 15 mM Imidazole, pH 8.0), and lysed by sonication in an ice-water bath. The suspensions were centrifuged (10,000 x g, 10 min) to pellet the insoluble matter, and the pellets were resuspended in denaturing binding buffer (8 M Urea) and centrifuged again. The supernatants were incubated on a rotary shaker with Ni-NTA agarose at 4°C for 60 min. Then, the columns were packed and washed with denaturing binding buffer and the fused peptides were eluted with elution buffer (40 mM Tris, 500 mM NaCl, 300 mM Imidazole, 8 M Urea, pH 8.0).

### Proteolytic cleavage and purification of recombinant peptides

Purified fused peptides in elution buffer (8 M Urea) were precipitated by rapid dilution with dilution buffer (40 mM Tris, pH 8.0) and collected by centrifugation at 5,000 × g for 10 min. The pellets were resuspended in cleavage buffer (20 mM Tris, 150 mM NaCl, pH 8.0, 0.3% sarkosyl). Thrombin was added to the samples and incubated at 23°C on a rotary shaker. Peptides were batch purified from the digestion product by affinity retention of the fusion partner on Ni-NTA agarose. Purified samples were stored at -20°C.

MALDI-TOF mass spectrometry was used to analyze the resultant peptide. Samples were diluted with formic acid 0.1% and methanol 3% and mixed with α-cyano-4-hydroxycinnamic acid (CHCA matrix). The spectrum was obtained in positive linear mode.

## Abbreviations

CPP: Cell penetrating peptide; KSI: Ketosteroid isomerase; Ni-NTA: Nickel-nitrilotriacetic acid; IMAC: Immobilized metal affinity chromatography; MALDI-TOF: Matrix assisted laser desorption ionization time-of-flight.

## Competing interests

The authors declare that they have no competing interests.

## Authors’ contributions

VR designed and carried out all of the experiments and participated in evaluation of the results and wrote the manuscript draft. BAA and JAA took part in designing the experiments, analyzing the results and writing the manuscript. All authors have read and approved the final manuscript.
